# Patient-reported outcomes following cemented versus cementless primary total knee arthroplasty: a comparative analysis based on propensity score matching

**DOI:** 10.1186/s12891-022-05899-1

**Published:** 2022-10-27

**Authors:** Hyunkyu Ko, Christopher E. Pelt, Brook I. Martin, The PEPPER Investigators, James A. Browne, Antonia F. Chen, Eric M. Cohen, Charles M. Davis, Navin D. Fernando, Kevin B. Fricka, Richard J. Friedman, Kevin L. Garvin, Richard Iorio, Michael S. Kain, Stephen L. Kates, Brent A. Lanting, Brock A. Lindsey, William J. Maloney, Robert M. Molloy, Michael A. Mont, Wayne E. Moschetti, James Nace, Charles L. Nelson, Kevin I. Perry, James D. Slover, Mark J. Spangehl, Lawrence M. Specht, Scott M. Sporer, Robert S. Sterling, Zeke J. Walton, Vincent D. Pellegrini

**Affiliations:** 1 Department of Orthopaedics, University Orthopaedic Center, University of Utah, Salt Lake City, UT USA; 2 Department of Orthopaedics, Dartmouth-Hitchcock Medical Center, Lebanon, NH USA

**Keywords:** Knee arthroplasty, Cemented TKA, Cementless TKA, Patient-Reported outcomes

## Abstract

**Background:**

Existing studies of patient-reported outcomes (PRO) following total knee arthroplasty (TKA) based on fixation methods (cemented vs cementless) are limited to single centers with small sample sizes. Using multicentered data,, we compared baseline and early post-operative global and condition-specific PROs between patients undergoing cemented versus cementless TKA.

**Methods:**

With PROs prospectively collected through Comparative Effectiveness Pulmonary Embolism Prevention After Hip and Knee Replacement (PEPPER) trial (ClinicalTrials.gov: NCT02810704), we examined pre- and post-operative (1, 3, and 6-months) outcomes in 5,961 patients undergoing primary TKA enrolled by 28 medical centers between December 2016 and August 2021. Outcomes included the short-form of the Knee Injury and Osteoarthritis Outcome Score (KOOS-Jr.), the Patient-Reported Outcomes Measurement Information System Physical Health (PROMIS-PH), and the Numeric Pain Rating Scale (NPRS). To minimize selection bias, we performed a 1-to-1 propensity score matched analysis to assess relative pre- to post-operative change in outcomes *within* and *between* cemented and cementless TKA groups.

**Results:**

With greater than 90% follow-up, significant pre to- post-operative improvements were observed in both groups. At 6 months, the cemented TKA group achieved a 3.3 point (55% of the Minimum Clinically Important Difference) greater improvement in the mean KOOS-Jr. (95%CI: 0.36, 6.30; *P* = 0.028) than did the cementless group with no significant between-group differences in PROMIS-PH and NPRS.

**Conclusions:**

In a large cohort of primary TKAs, patients with cemented fixation reported early incremental benefit in KOOS-Jr. over those with cementless TKA. Future studies are warranted to capture longer follow-up of PROs.

**Supplementary Information:**

The online version contains supplementary material available at 10.1186/s12891-022-05899-1.

## Background

There has been a large increase in the rate of total knee arthroplasty (TKA) over the past decade in the United States. Among the Medicare population, roughly 351,000 TKA were performed in 2010, more than doubling to 738,000 in 2020 [1]. TKA provides relief of pain and recovery of functional status for patients with knee osteoarthritis [2, 3]. Two types of fixation of TKA implants can be utilized, with either the use of polymethyl-methacrylate (PMMA) bone cement or cementless implants which most often utilize a porous or textured surface to promote direct osseous integration of the implant.

Previous studies have attempted to determine the superiority of these two implant fixation methods. Based on early studies demonstrating their superiority, cemented TKA has been widely used in the United States as the gold standard [4–7]. However, recent papers have challenged the superiority of cement. Some show that there are no statistically significant differences in clinical and cost outcomes [8–16], while others suggest that cementless TKA achieves greater survivorship and long-term functional recovery [17, 18] and lower procedure cost [19]. While cement provides immediate fixation, cementless fixation requires the biologic process of osseous integration to occur, and some reports show higher early rates of subtle radiolucencies, implant migration, aseptic loosening and higher early pain scores in cementless TKA [6, 20–23]. Because of the importance of pain relief and early function to our patients following TKA [24], the relevance of early outcomes following TKA should not be underestimated.

Lacking from the current body of evidence are rigorous comparisons of pre-and early post-operative patient-reported measures of pain and functional disability from a large multicenter cohort. Researchers have only recently focused on patient-reported outcomes (PRO) as a valuable tool to assess patient recovery, pain and function following TKA [12–18, 25, 26]. With few exceptions [18, 26], most studies have found no statistically significant differences in PROs between cemented and cementless TKA [12–17, 25]. However, these studies have been focused on the experience of a single surgeon or single institution, with limited generalizability. Further, many prior studies cited in the literature compare outcomes of older cemented and cementless TKA technologies [27], and recent design improvements with more modern designs remain to be investigated in a contemporary dataset.

In order to improve the understanding of early patient recovery after TKA, we analyzed global and condition-specific PROs between patients who received cemented TKA compared with cementless implants using prospectively collected data from the multicenter Patient-Centered Outcomes Research Institute (PCORI) funded Comparative Effectiveness of Pulmonary Embolism Prevention after Hip and Knee Replacement (PEPPER) trial.

## Methods

### Study design

PROs were prospectively collected through the PEPPER trial (NCT02810704 at ClinicalTrials.gov), a large, multi-centered, randomized clinical trial evaluating safety and effectiveness of prophylactic antithrombotic medication after total hip and knee arthroplasty [28]. A central (single) human subjects review board from the Medical University of South Carolina provided regulatory review of the study (Approval Number: Pro00053742). All procedures were performed in accordance with the ethical standards of the institution.

While PEPPER is an ongoing randomized trial for antithrombotic drug safety, assignment to cement or cementless TKA was at the discretion of participating surgeons. Furthermore, the primary safety outcomes of venous thromboembolism, bleeding, and death in PEPPER are currently blinded until the trial ends due to data integrity protocols, and as such, are not available for our analysis. Nevertheless, PEPPER provides data on 6 month longitudinal PROs (global and condition-specific), patient characteristics (age, Body Mass Index (BMI), gender, race, ethnicity, education, working status, drinking, smoking, and comorbidity), and implant fixation methods (cemented and cementless), collected from study participants enrolled by 28 North American hospitals (among a total of 31 participant centers). This enabled us to analyze prospectively collected data to evaluate the influence of implant fixation method of TKA on short term PROs. Specifically, we included participants enrolled in the PEPPER trial between 12/19/2016 and 8/31/2021 and performed 1-to-1 propensity score matched analysis to compare outcomes between cemented and cementless TKA.

### Data outcomes

**Fig. 1:**
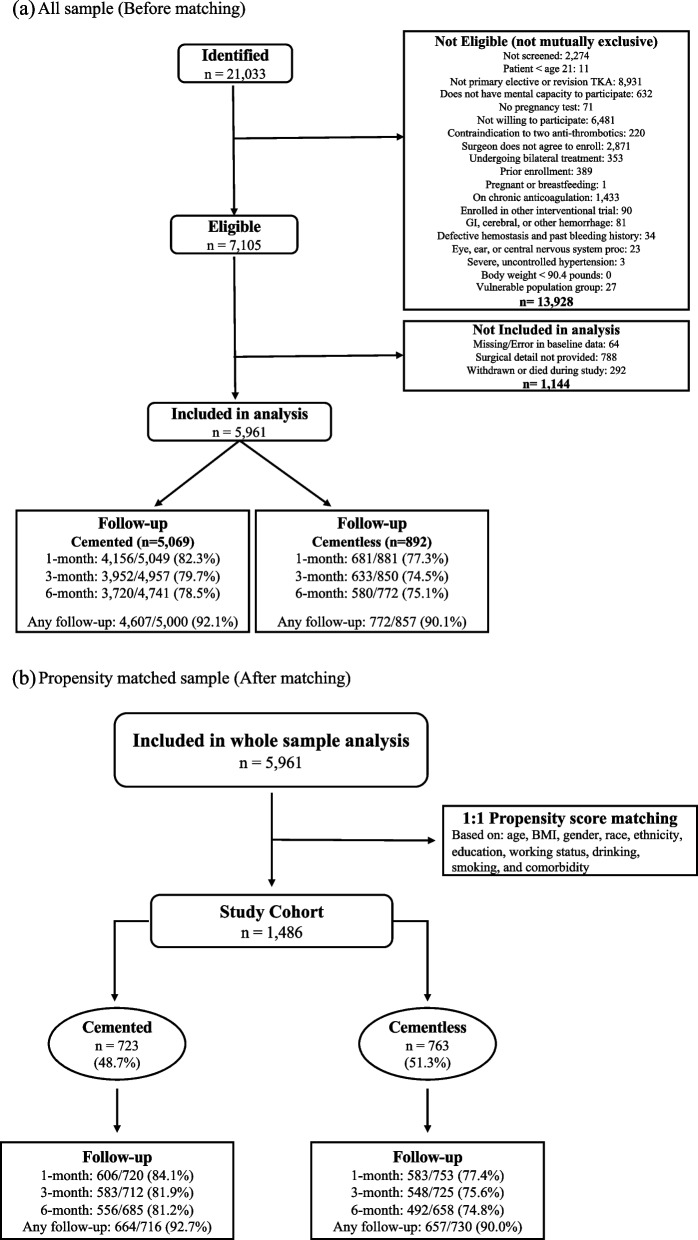
Study cohort and follow-up by implant fixation methods (**a**) All sample (Before matching) Note: PROs were collected at 1-month, 3-month, and 6-month using centralized telephone interviews, web-based surveys, and postage-paid reply mail surveys. (**b**) Propensity matched sample (After matching) Note: PROs were collected at 1-month, 3-month, and 6-month using centralized telephone interviews, web-based surveys, and postage-paid reply mail surveys

We included adult patients undergoing elective primary TKA who provided an informed consent, excluding those on chronic anticoagulation or who had comorbidities that confounded the assessment of antithrombotic medication safety. We categorized patients by two treatment subgroups, which were utilized at the discretion of the surgeon: (1) cemented TKA and (2) cementless TKA. Even though PEPPER enrolled subjects with knee revision or unicompartmental knee replacement procedures, they were excluded from our analysis in order to focus on the question of cemented vs cementless fixation in a more homogenous cohort of primary TKA. We also excluded those who died or withdrew from the study because they lacked complete PRO’s. Detailed inclusion and exclusion criteria for study participation can be found in Fig. 1-(a).

Using centralized telephone interviews, web-based surveys, and postage-paid reply mail surveys, PROs were collected pre-operatively and at 1-month (37 days post-operative, -7/ + 10 days), 3-month (90 days post-operative, -10/ + 14 days), and 6-months (180 days post-operative, -28/ + 28 days). Patients were contacted for follow-up regardless of complication, or if they changed or discontinued antithrombotic medications. In addition to PROs, patient demographic information and comorbidities were collected preoperatively, and operative characteristics, including the use of cement, were specifically reported by the enrolling center.

Primary outcomes include validated measure of patient-reported knee function, general physical health, and pain. Knee function was evaluated using the short form version of the Knee Injury and Osteoarthritis Outcome Score (KOOS-Jr.), which contains 7 questions to assess pain, function, and quality of daily living within the past week [29]. The KOOS-Jr. is scored on a 0–100 scale with a larger value representing a higher knee function [30]. The Patient-Reported Outcomes Measurement Information System Physical Health (PROMIS-PH) Summary was used to assess global pain and general (physical) health. The PROMIS-PH is a 10-item measure, calculated using a T-score in which distributions are standardized with a mean (standard deviation) of 50 (10), with greater scores indicating better health [31]. The Numeric Pain Rating Scale (NPRS) is scored on a 0–10 scale with a lower score representing less pain [32]. We also report the proportion of patients who achieved a Minimum Clinically Important Difference (MCID) using threshold for KOOS-Jr., PROMIS-PH, and NPRS of 6, 7.9, and 2 points respectively [30, 32].

### Statistical analysis

One of the challenges in comparing PROs between cemented and cementless TKA is that even after adjusting for observable characteristics of patients, there might still be confounders that lead to biased estimates of the association between implant fixation methods and PROs. For example, if many surgeons tend to use cementless fixation in younger patients and use cemented fixation in older patients due to the fact that younger patients are more likely to have better bone quality, then there would be a concern for potential bias due to extrapolation even after controlling for age. Also, if surgeons’ motivation and surgical skills, which are hard to observe, are correlated with decision on cemented/cementless fixation, accounting for observable characteristics could not help reduce bias.

To minimize these biases, we conducted a propensity score matched analysis. Specifically, we employed single nearest-neighbor matching (also known as 1-to-1 matching) in which each cementless case is matched to a cemented participant that most closely resembles their observed patient characteristics. We matched cases to control based on age, BMI, gender, race, ethnicity, education, working status, drinking, smoking, and comorbidity. Participants who are outside the “region of common support” were dropped from the analysis. There are several reasons why we used 1-to-1 matching. First, on average, discordant matches can lead to biased estimates in multiple nearest neighbor matching than in 1-to-1 matching [33]. Despite excluding unmatched observations, the overall power is not compromised because the improved quality of matches from 1-to-1 matching can lead to increased power [34]. Based on our propensity matched sample size, our analysis was sufficiently powered to detect a 2.0 point between-group difference in KOOS-Jr., a 1.2-point difference in PROMIS-PH, and a 0.35-point difference in NPRS, with at least 85% power. These differences compare favorably with the previously mentioned MCIDs for each measure. We also imposed a replacement as well as a caliper (0.25 standard deviation) to increase the average quality of matches, further reducing bias [35]. We estimated the Average Treatment Effect (ATE) for the sample within the range of common support [36]. Stata-17.0-MP (StataCorp, College Station, TX) was used to conduct all analyses.

## Results

### Study population

Among 5,961 TKA patients who were eligible for this analysis, there were 5,069 (85.0%) cemented and 892 (15.0%) cementless procedures. Patients undergoing bilateral procedures or second side surgery were excluded from PEPPER. Before constructing a propensity score matched sample, patients with cemented TKA had a lower BMI, were more likely to be white, non-Hispanic, and college graduates. They were also more likely to be a former smoker compared to patients with cementless TKA (Appendix A1). Excluded cases who died or withdrew from the study were older (66.1 compared to 64.2; *p* < 0.001) and more likely to be white (89.2% compared to 77.9%; *p* = 0.021) compared to those included in the analysis.

**Fig. 2:**
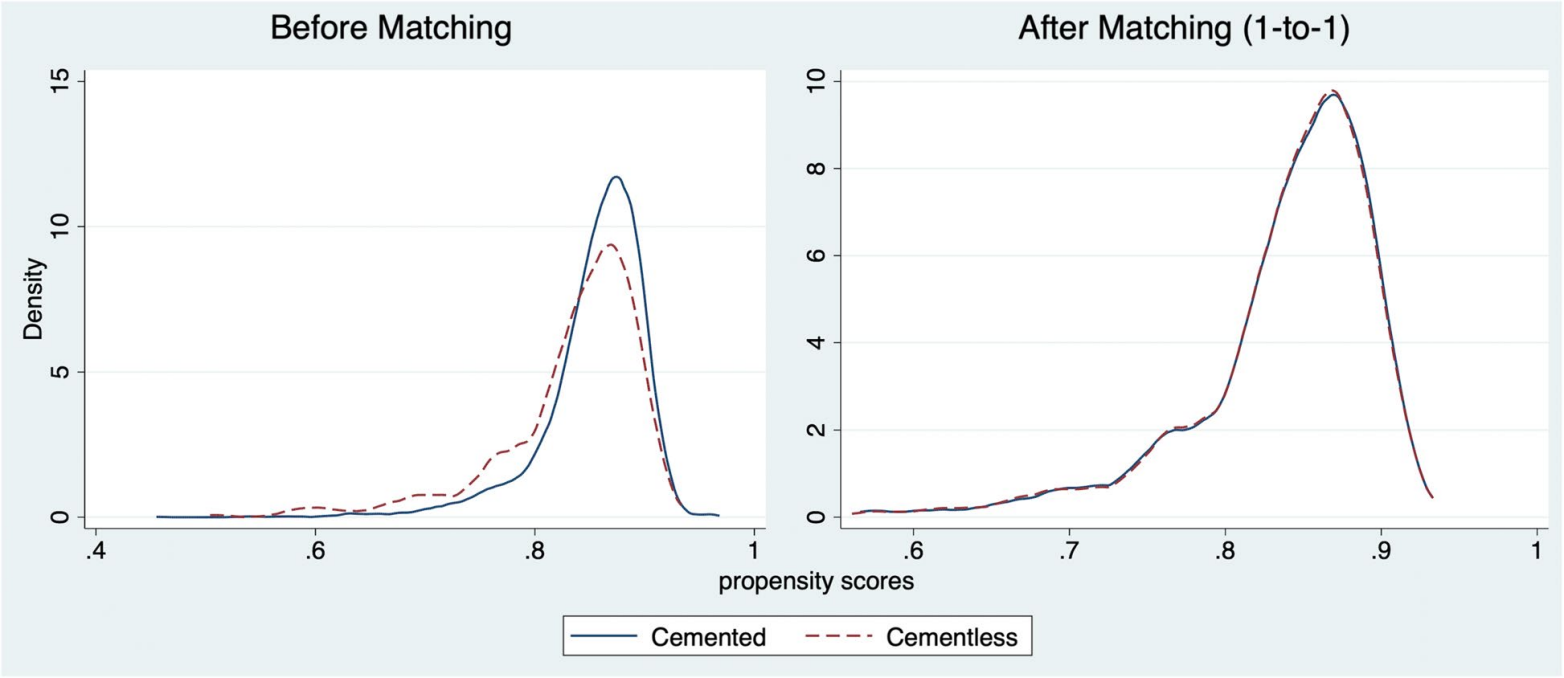
Balance plot of study cohort before and after the propensity score matching. Note: Distribution of the propensity scores before and after 1:1 matching between cemented and cementless TKA groups

Following propensity-matching, the sample size reduced to 1,486 (cemented: 723, cementless: 763). There were no significant differences in observable patient characteristics or comorbidities between cemented and cementless TKA (Table 1). The distributions of estimated propensity scores for both groups before and after matching confirm that our propensity score matching approach achieved balance between groups with respect to observed covariates (Fig. 2).

**Table 1:** Descriptive statistics from Propensity matched sample (1:1 matching)

Baseline Characteristic	(1)	(2)	(3)	(4)
	**Cemented**	**Cementless**	**All**	***p***-value
AGE				
Age (mean)	64.5	64.3	64.4	0.691
Body Mass Index (BMI)				
BMI (mean)	33.1	33.2	33.1	0.731
Gender				
Female (%)	57.7	56.1	56.9	0.538
Male (%)	42.3	43.9	43.1	
Race				
White (%)	78.7	76.3	77.5	0.536
Black (%)	13.6	15.1	14.3	
Other/Multiple (%)	7.7	8.6	8.2	
Ethnicity				
Hispanic (%)	4.6	3.7	4.1	0.385
Non-Hispanic (%)	95.4	96.3	95.9	
Education				
Less than college (%)	60.2	64.5	62.4	0.086
College graduate (%)	39.8	35.5	37.6	
Work				
Working (%)	36.2	36.6	36.4	0.877
Unemployed (%)	2.2	2.1	2.2	
Sick leave or maternity leave (%)	14.3	15.6	14.9	
Disabled due to hip or knee pain (%)	47.3	45.7	46.5	
Alcohol				
Never (%)	29.3	31.3	30.4	0.614
Monthly or less (%)	26.0	26.1	26.0	
2–4 times a month (%)	16.1	16.4	16.2	
2–3 times a week (%)	14.5	14.8	14.7	
4 or more times a week (%)	14.1	11.4	12.7	
Smoke				
Never (%)	58.9	58.6	58.8	0.981
Current (%)	7.3	7.2	7.3	
Former (%)	33.8	34.2	33.9	
Comorbidity count				
0 (%)	60.0	59.0	59.5	0.589
1 (%)	25.9	28.1	27.0	
2 + (%)	14.1	12.9	13.5	
N (% of total)	723 (48.7)	763 (51.3)	1,486	

Note: Descriptive statistics are from 1 to 1 nearest matching within caliper (0.25 SD)

Significance tests were based on Chi-square comparisons for categorical variables and based on analysis of variance (ANOVA) for continuous variables

Among the matched cohort, 91.3% of patients who passed through the 6-month follow-up window at the time of analysis (92.7% for cemented TKA; 90.0% for cementless TKA) completed at least one post-operative PRO collection survey (at either 1-month, 3-months, or 6-months.). Follow-up rates were similar between the two groups of patients (Fig. 1-(b)). The baseline characteristic for ethnicity was the most frequently unanswered response, but was only missing in less than 1% of cases.

### The effects of TKA on PROs in the propensity matched sample

**Fig. 3:**
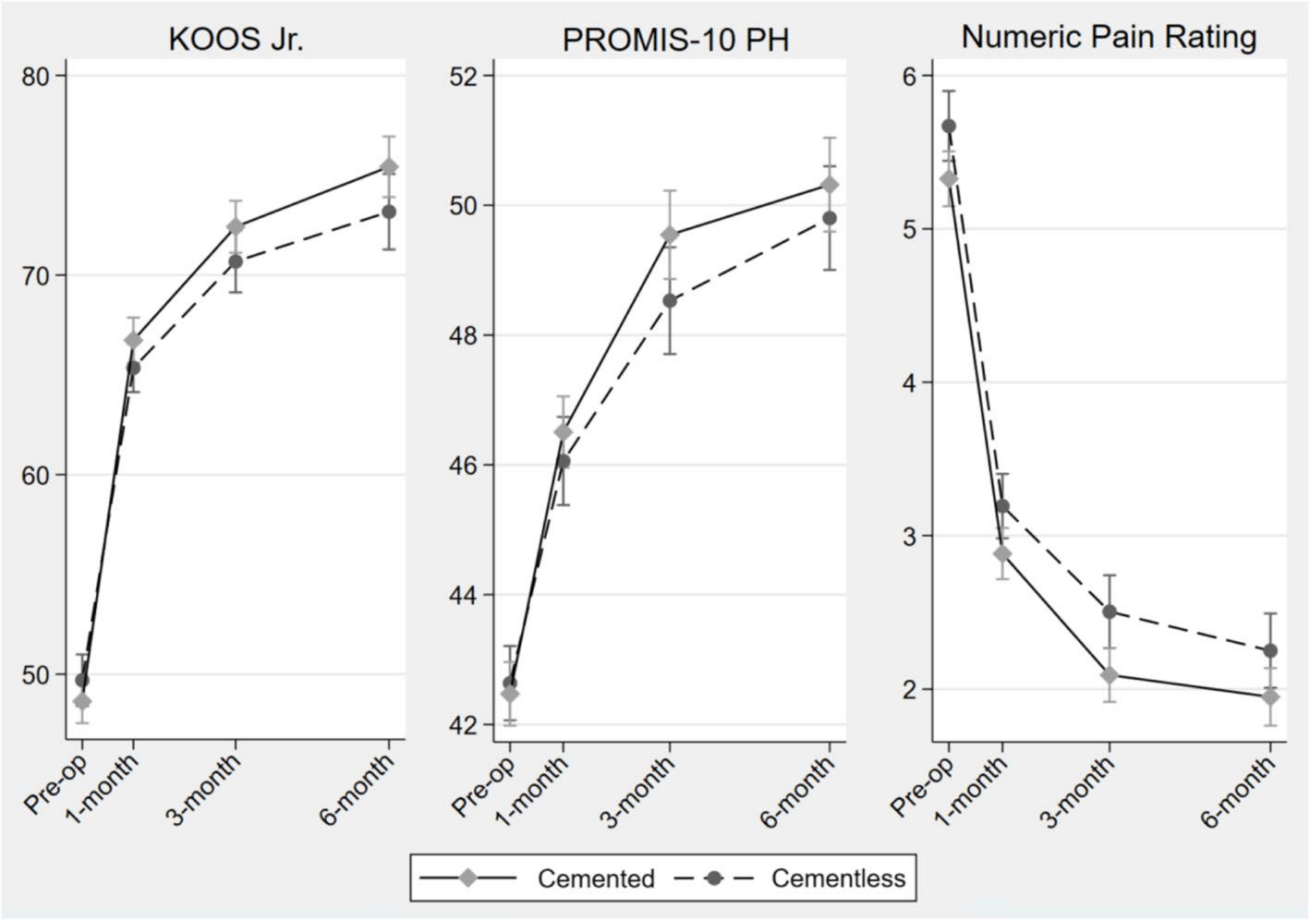
PROs following TKA between cemented and cementless fixation in the propensity-matched cohort. Note: Patient-reported outcomes following cemented and cementless total knee arthroplasty. Estimated coefficients were adjusted for age, BMI, gender, race, ethnicity, education, working status, drinking, smoking, and comorbidity

Overall, for both fixation methods, there were statistically significant pre- to post-operative improvements in mean knee function, physical health, and pain by 6-months (Fig. 3). Specifically, the mean adjusted KOOS-Jr., PROMIS-PH, and NPRS improved by 26.2 (95%CI: 24.9, 27.4), 7.7 (95%CI: 7.1, 8.2), and -3.3 (95%CI: -3.5, -3.1) points in the cemented TKA and by 25.3 (95%CI: 24.0, 26.6), 7.4 (95%CI: 6.8, 8.0), and -3.5 (95%CI: -3.7, -3.3) points in the cementless group, respectively (Table 2). Both Table 2 and Fig. 3 show that improvements increased over time, with the largest improvement appearing from pre-operative to 1 month, and slower improvements at 3 and 6 months, suggesting stabilization of the effects of TKA on PROs over time.

**Table 2:** Adjusted Mean Patient-Reported Outcomes and Improvements by Month (1:1 matching)

*Total Knee Arthroplasty*		*Cemented*			*Cementless*		
		N	Mean	95% CI	*N*	Mean	95% CI
KOOS-Jr	Baseline	723	48.9	(47.8 – 50.0)	763	48.7	(47.5 – 49.8)
	1-month	723	66.6	(65.4 – 67.8)	763	65.0	(63.7 – 66.2)
	3-month	723	72.3	(71.1 – 73.6)	763	70.9	(69.6 – 72.2)
	6-month	723	75.1	(73.8 – 76.3)	763	73.9	(72.6 – 75.3)
	1-mon-Base	723	17.7	(16.5 – 18.9)	763	16.3	(15.1 – 17.5)
	3-mon-Base	723	23.4	(22.1 – 24.6)	763	22.3	(21.0 – 23.5)
	6-mon-Base	723	26.2	(24.9 – 27.4)	763	25.3	(24.0 – 26.6)
PROMIS-PH	Baseline	723	42.6	(42.0 – 43.2)	763	42.2	(41.6 – 42.9)
	1-month	723	46.5	(45.9 – 47.1)	763	45.8	(45.1 – 46.4)
	3-month	723	49.6	(49.0 – 50.2)	763	48.4	(47.7 – 49.1)
	6-month	723	50.2	(49.6 – 50.9)	763	49.6	(49.0 – 50.3)
	1-mon-Base	723	3.9	(3.4 – 4.5)	763	3.5	(3.0 – 4.0)
	3-mon-Base	723	7.0	(6.5 – 7.6)	763	6.2	(5.6 – 6.7)
	6-mon-Base	723	7.7	(7.1 – 8.2)	763	7.4	(6.8 – 8.0)
NPRS	Baseline	723	5.3	(5.1 – 5.5)	763	5.7	(5.6 – 5.9)
	1-month	723	2.9	(2.7 – 3.1)	763	3.2	(3.0 – 3.4)
	3-month	723	2.1	(1.9 – 2.3)	763	2.5	(2.3 – 2.7)
	6-month	723	2.0	(1.8 – 2.2)	763	2.3	(2.1 – 2.5)
	1-mon-Base*	723	-2.4	(-2.6 – -2.2)	763	-2.5	(-2.7 – -2.3)
	3-mon-Base*	723	-3.2	(-3.4 – -3.0)	763	-3.2	(-3.4 – -3.0)
	6-mon-Base*	723	-3.3	(-3.5 – -3.1)	763	-3.5	(-3.7 – -3.3)

^*^ Lower scores are better, so that negative signs represent improvement

Note: Baseline, 1-month, 3-month, and 6-month represent absolute score at each time point, while 1-mon-Base, 3mon-Base, and 6-mon-Base represent changes at1,3,6 months from baseline

### PROs between cemented and cementless fixation in TKA

We compared pre-operative to post-operative changes in PROs between cemented and cementless TKA using a propensity score matched analysis (Table 3). Only the observed differences in the mean adjusted KOOS-Jr. were statistically significant between groups, while no statistically significant differences were found between groups in the adjusted measures for PROMIS-PH and NPRS at any time point. Specifically, at 6 months, cemented TKA achieved a 3.3 point greater improvement in KOOS-Jr. (95%CI: 0.36, 6.30; *P* = 0.028) compared to cementless TKA (Table 3).

**Table 3:** Results from Propensity Score Matching (1-to-1 matching)

***Outcomes***	*KOOS-Jr*		*PROMIS-PH*		*NPRS*	
	$\beta$	*P*	$\beta$	*P*	$\beta$	*P*
Surgical approach (ref = cementless)						
Cemented	-1.37	.113	-0.11	.766	-0.34	.022
Follow-up (ref = baseline)						
1-month	15.7	.000	3.43	.000	-2.48	.000
3-month	21.0	.000	5.90	.000	-3.17	.000
6-month	23.5	.000	7.17	.000	-3.42	.000
Approach interaction (ref = cementless)						
Cemented *1-month	2.46	.042	0.61	.303	0.04	.862
Cemented *3-month	2.80	.037	1.19	.076	-0.07	.740
Cemented *6-month	3.33	.028	0.69	.302	0.05	.836
AGE						
Age (mean)	0.27	.000	0.09	.000	-0.03	.000
Sex (ref = male)						
Female	-1.48	.023	-1.09	.001	0.20	.058
Body Mass Index	-0.08	.091	-0.16	.000	0.002	.813
Race (ref = white)						
Black	-3.41	.004	-1.86	.000	1.11	.000
Other or multiple	-1.39	.271	-1.91	.000	0.58	.001
Hispanic (ref = no)						
Yes	2.51	.106	1.28	.064	0.43	.077
Education (ref = less than college)						
College or higher	0.61	.392	0.93	.008	-0.20	.074
Working status (ref = working)						
Unemployed	-0.68	.728	-0.81	.337	0.03	.945
Sick leave or maternity leave	-2.69	.036	-4.13	.000	0.90	.000
Disabled due to hip or knee pain	0.55	.497	-0.84	.038	-0.02	.904
Alcohol use (ref = never)						
Monthly or less	-1.10	.227	0.52	.213	0.06	.652
2–4 times a month	-2.11	.061	0.48	.329	0.04	.798
2–3 times a week	-0.01	.995	1.64	.000	-0.19	.229
4 or more times a week	0.79	.502	1.72	.001	-0.25	.135
Smoke (ref = never)						
Current	-3.69	.029	-1.43	.078	0.59	.015
Former	-0.57	.411	-1.00	.003	0.11	.339
Comorbidity (ref = no)						
COPD	-2.18	.152	-1.54	.009	0.07	.746
Paralysis	15.9	.028	15.2	.000	-1.69	.173
Heart attack	0.22	.863	-0.09	.888	0.36	.149
Carotid artery disease	-1.28	.621	-0.24	.858	-0.23	.626
Stroke	2.00	.279	0.53	.507	-1.60	.680
Rheumatoid arthritis	-1.73	.243	-1.64	.004	0.41	.038
Diabetes	1.98	.022	-0.72	.108	0.03	.827
Cancer	1.46	.210	0.45	.374	-0.11	.493
Liver disease	-0.34	.876	0.66	.507	-0.48	.142
Peripheral vascular disease	3.13	.133	-1.26	.192	-0.48	.149
Kidney disease	-6.15	.000	-4.04	.000	0.53	.077
Ulcer disease	-6.90	.007	-2.92	.000	0.81	.023
HIV or AIDS	-5.50	.289	-4.44	.084	1.12	.194
Constant	37.3	.000	43.5	.000	7.17	.000

Note: The estimated coefficients were from 1 to 1 nearest matching within caliper (0.25 SD)

**Fig. 4:**
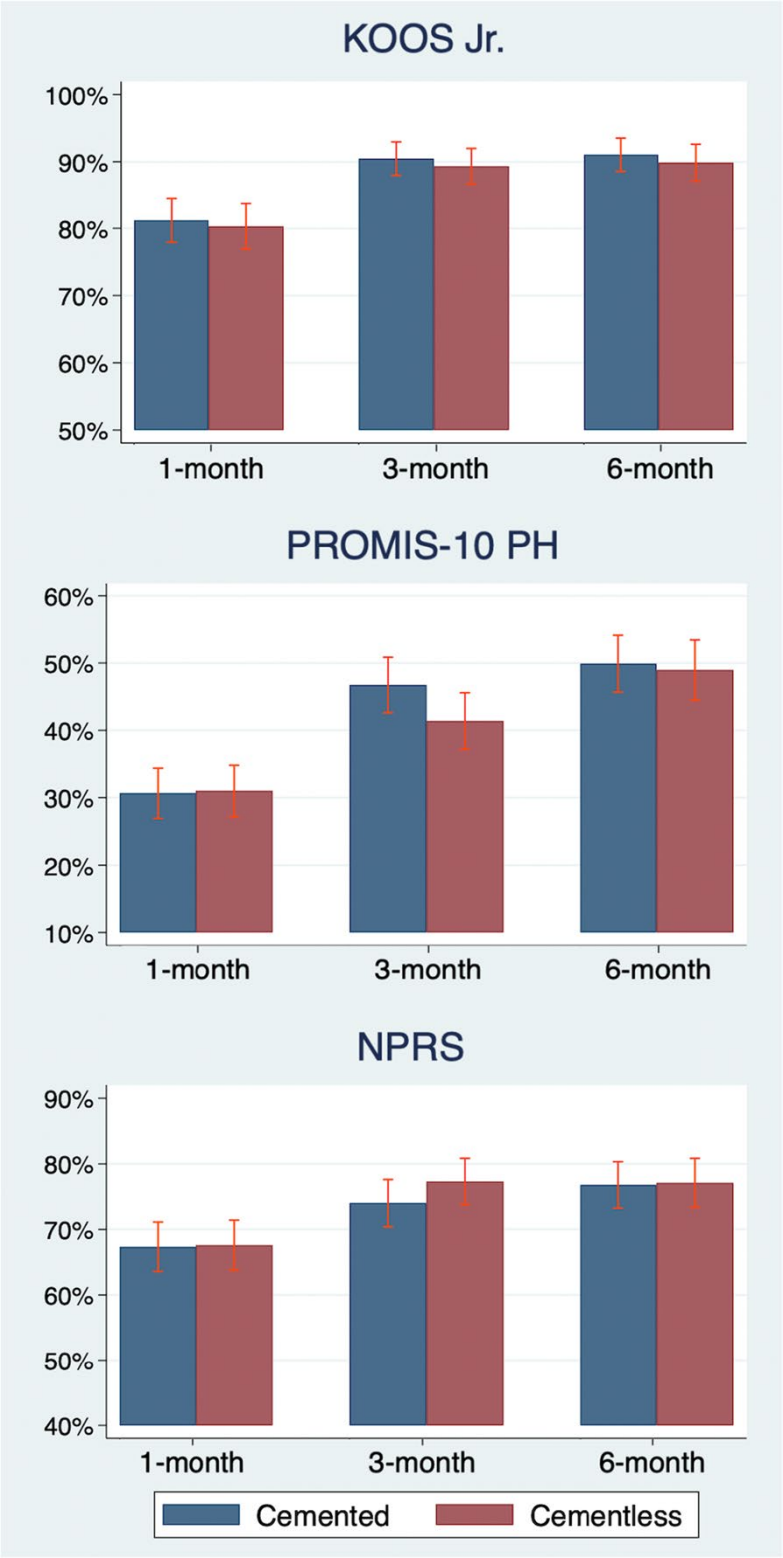
Proportion of patients achieving MCID at 1, 3, and 6 months among the propensity-matched cohort. Note: Proportion of patients within each group (cemented vs cementless) who achieved the Minimum Clinically Important Difference (MCID) in each patient-reported outcomes (KOOS Jr., PROMIS-10 PH, and NPRS)

A responder analysis shows the proportion of patients within each group who achieved the MCID in each outcome over time (Fig. 4). Overall, the proportion of patients who achieved the MCID increased over time. By 6 months, 90.5%, 49.5%, and 76.9% of patients who underwent TKA achieved the MCID in KOOS-Jr., PROMIS-PH, and NPRS respectively, with no statistically significant differences in the proportion of patients who achieved the MCID between cemented and cementless groups in each time point.

### Comparison between propensity matched sample and whole sample

Between-group effects in the PROMIS-PH or NPRS were similar for the propensity matched sample and the entire unmatched sample (based on a linear mixed-effects regression). However, the magnitude of the effect coefficients for KOOS-Jr. were decreased by half in the entire, unmatched sample (Appendix A2,) but did not change the overall trend of the results or conclusions. For example, in both samples, the differences in KOOS-Jr. between cemented and cementless TKA were mostly significant and increased over time. This suggests a low likelihood of bias after adjusting for observable characteristics.

## Discussion

Our study demonstrated significant improvements in patient-reported pain and functional outcomes among all patients enrolled in the PEPPER trial who underwent either cemented or cementless TKA. Patients who underwent cemented TKA had a statistically significant greater improvement in knee function scores (KOOS-Jr.) than did those having a cementless procedure, but the effect difference (3.3 points; 55% of the MCID) was small from a clinical perspective. There were no other significant relative differences in general physical health or pain based on implant fixation methods. While group-mean differences in specific knee function did not reach MCID, the findings that patients undergoing cemented TKA achieved better improvement in KOOS-Jr. compared to those with cementless TKA may be of substantial importance to both patients and surgeons in discussing expectations for early recovery outcomes. Surgeons and patients may appropriately use this information to inform pre-operative counseling and shared decision-making.

The finding of a between-group treatment effect less than the MCID in the present study broadly aligns with several smaller studies from single centers that found no or little significant differences in PROs between cemented and cementless TKA [12–17, 25, 26]. A notable exception is the meta-analysis by Liu et al. [18] which reported better long term functional benefit for cementless patients (mean difference: 1.70; *p* = 0.004). Their finding was based on the Knee Society function scores of 652 subjects in cementless TKA and 656 subjects in cemented TKA from 9 studies with at least 2-year of follow-up. Notably, our analysis was based on larger overall sample size with a propensity-matched sample that still exceeded that the size of that included in the studies analyzed by Liu et al. Specifically, in our study, longitudinal PROs were collected prospectively from a large cohort of TKA patients enrolled by 28 medical centers among 31 total centers participating in PEPPER. This larger sample size enabled us to perform a propensity-matched analysis, which helps address any selection-by-indication bias, and increased external validity (generality of findings). In addition, our study focused on commonly used and validated instruments for both global and condition-specific measures.

This study has several limitations. First, while PROs were prospectively collected from the PEPPER trial, it is important to acknowledge that the randomization in the PEPPER trial is unrelated to our analysis; specifically, patients were not randomized to receive cemented or cementless TKA. Even though we employed a propensity score matching approach to reduce selection-by-indication bias, we cannot fully discount the potential for unobserved confounders that could weaken the validity of our findings. Second, because PEPPER is focused on safety surrounding anti-thrombotic drugs, long-term PRO’s (beyond the 6-month window) were not available. While it is known that most improvement in PROs after TKA occurs in the first 3 months, and PROs tend to stabilize after 6 months [37, 38], the extrapolation of our finding of a higher KOOS Jr after cemented compared with cementless might not be sustained in the longer term. PEPPER patients were enrolled by multiple (mostly academic) institutions with highly developed research and clinical trials infrastructures, which may limit our generalizability to other settings. Differences in safety, such as antithrombotic events, infections, or readmissions were not available for this analysis because they remain blinded until the PEPPER trial has been completed. The PEPPER trial did not collect all potentially confounding clinical details such as the type of TKA implant, kinematic alignment, type of porous metal coating, use of robot surgery. The degree to which these factors are disproportionately related to patient reported outcome is unknown and are thus a potential source of unobserved confounding. Lastly, although single nearest-neighbor matching approach could increase quality of matches, it also increased the variance of sample estimates by dropping many observations, which may lead to less precise parameter estimates.

## Conclusion

While further studies with longer-term follow-up of PROs are required to definitively compare outcomes between cemented and cementless TKA, our findings suggest that cemented TKA potentially affords a slight benefit in patient-reported physical function during the first 6 months following surgery. Such an early advantage in physical function after cemented TKA could provide a benefit to patient pain, function, morale, and satisfaction following a major surgical procedure and may represent an important addition to pre-operative patient counseling and shared decision-making prior to total knee replacement.

## Supplementary Information

**Additional file 1: Appendix A1.** Descriptive statistics from all sample **Appendix A2.** Results for regression models from all sample (Mixed effects).

## Data Availability

The data that support the findings of this study are available from Vincent D. Pellegrini, Professor of Orthopaedics, Dartmouth-Hitchcock Medical Center but restrictions apply to the availability of these data, which were used under license for the current study, and so are not publicly available. Data are however available from the authors upon reasonable request and with permission of Vincent D. Pellegrini, Professor of Orthopaedics, Dartmouth-Hitchcock Medical Center.

## Abbreviations

PRO: Patient-reported outcomes
TKA: Total knee arthroplasty
PEPPER: Comparative Effectiveness of Pulmonary Embolism Prevention after Hip and Knee Replacement
KOOS-Jr.: Short-form of the Knee Injury and Osteoarthritis Outcome Score
PROMIS-PH: Patient-Reported Outcomes Measurement Information System Physical Health
NPRS: Numeric Pain Rating Scale
PMMA: Polymethyl-methacrylate
PCORI: Patient-Centered Outcomes Research Institute
BMI: Body Mass Index
MCID: Minimum Clinically Important Difference
ATE: Average Treatment Effect
