# Supplementary material for: Patient-reported outcomes following cemented versus cementless primary total knee arthroplasty: a comparative analysis based on propensity score matching
Source: BMC Musculoskelet Disord. 2022 Oct 27;23:934. doi: 10.1186/s12891-022-05899-1 (PMC9608915; doi:10.1186/s12891-022-05899-1)
Supplement: Supplementary file 1 — Additional file 1: Appendix A1. Descriptive statistics from all sample Appendix A2. Results for regression models from all sample (Mixed effects). [file 12891_2022_5899_MOESM1_ESM.docx]

APPENDIX A1

Descriptive statistics from all sample

| **Baseline Characteristic** | **(1)** | **(2)** | **(3)** | **(4)** |
| --- | --- | --- | --- | --- |
|  | **Cemented** | **Cementless** | **All** | **p-value** |
| AGE |  |  |  |  |
| Age (mean) | 64.2 | 64.4 | 64.2 | 0.500 |
| Body Mass Index (BMI) |  |  |  |  |
| BMI (mean) | 32.7 | 33.2 | 32.8 | 0.027 |
| Gender |  |  |  |  |
| Female (%) | 61.3 | 56.7 | 60.6 | 0.010 |
| Male (%) | 38.7 | 43.3 | 39.4 |  |
| Race |  |  |  |  |
| White (%) | 78.5 | 74.2 | 77.9 | <0.001 |
| Black (%) | 16.7 | 15.3 | 16.5 |  |
| Other/Multiple (%) | 4.8 | 10.5 | 5.6 |  |
| Ethnicity |  |  |  |  |
| Hispanic (%) | 2.5 | 4.9 | 2.9 | <0.001 |
| Non-Hispanic (%) | 97.5 | 95.1 | 97.1 |  |
| Education |  |  |  |  |
| Less than college (%) | 59.6 | 64.2 | 60.3 | 0.008 |
| College graduate (%) | 40.4 | 35.8 | 39.7 |  |
| Work |  |  |  |  |
| Working (%) | 39.2 | 36.1 | 38.7 | 0.226 |
| Unemployed (%) | 1.9 | 2.0 | 1.9 |  |
| Sick leave or maternity leave (%) | 16.2 | 15.6 | 16.1 |  |
| Disabled due to hip or knee pain (%) | 42.7 | 46.3 | 43.3 |  |
| Alcohol |  |  |  |  |
| Never (%) | 32.4 | 32.4 | 32.4 | 0.415 |
| Monthly or less (%) | 26.9 | 25.8 | 26.7 |  |
| 2-4 times a month (%) | 15.6 | 15.3 | 15.5 |  |
| 2-3 times a week (%) | 13.1 | 15.4 | 13.5 |  |
| 4 or more times a week (%) | 12.0 | 11.1 | 11.9 |  |
| Smoke |  |  |  |  |
| Never (%) | 56.8 | 58.7 | 57.1 | 0.004 |
| Current (%) | 5.1 | 7.3 | 5.4 |  |
| Former (%) | 38.1 | 34.0 | 37.5 |  |
| Comorbidity count |  |  |  |  |
| 0 (%) | 59.4 | 59.0 | 59.4 | 0.147 |
| 1 (%) | 27.5 | 28.4 | 27.6 |  |
| 2+ (%) | 13.1 | 12.6 | 13.0 |  |
| N (% of total) | 5,069 (85.0) | 892 (15.0) | 5,961 |  |

Significance tests were based on Chi-square comparisons for categorical variables and based on analysis of variance (ANOVA) for continuous variables.

APPENDIX A2

Results for regression models from all sample (Mixed effects)

| ***Outcomes*** | *KOOS-Jr* | | *PROMIS-PH* | | *NPRS* | |
| --- | --- | --- | --- | --- | --- | --- |
|  | $\beta$ | *P* | $\beta$ | *P* | $\beta$ | *P* |
| Surgical approach (ref = cementless) |  |  |  |  |  |  |
| Cemented | -0.70 | .232 | -0.14 | .644 | -0.18 | .045 |
| Follow-up (ref = baseline) |  |  |  |  |  |  |
| 1-month | 16.2 | .000 | 3.49 | .000 | -2.50 | .000 |
| 3-month | 22.4 | .000 | 6.30 | .000 | -3.23 | .000 |
| 6-month | 25.2 | .000 | 7.43 | .000 | -3.46 | .000 |
| Approach interaction (ref = cementless) |  |  |  |  |  |  |
| Cemented *1-month | 1.11 | .093 | 0.23 | .415 | 0.09 | .369 |
| Cemented *3-month | 1.40 | .035 | 0.47 | .105 | -0.02 | .880 |
| Cemented *6-month | 1.63 | .017 | 0.09 | .765 | 0.06 | .591 |
| Age | 0.22 | .000 | 0.08 | .000 | -0.02 | .000 |
| Sex (ref = male) |  |  |  |  |  |  |
| Female | -1.14 | .000 | -1.06 | .000 | 0.25 | .000 |
| Body Mass Index | -0.04 | .080 | -0.13 | .000 | -0.001 | .883 |
| Race (ref = white) |  |  |  |  |  |  |
| Black | -3.88 | .000 | -1.03 | .000 | 0.90 | .000 |
| Other or multiple | -1.34 | .049 | -0.61 | .091 | 0.24 | .016 |
| Hispanic (ref = no) |  |  |  |  |  |  |
| Yes | -1.05 | .266 | -0.76 | .127 | 0.73 | .000 |
| Education (ref = less than college) |  |  |  |  |  |  |
| College or higher | 1.06 | .001 | 1.10 | .000 | -0.31 | .000 |
| Working status (ref = working) |  |  |  |  |  |  |
| Unemployed | -1.18 | .291 | -1.75 | .002 | 0.41 | .010 |
| Sick leave or maternity leave | -4.57 | .000 | -4.35 | .000 | 0.93 | .000 |
| Disabled due to hip or knee pain | -0.44 | .227 | -0.81 | .000 | 0.06 | .251 |
| Alcohol use (ref = never) |  |  |  |  |  |  |
| Monthly or less | 0.04 | .916 | 0.37 | .069 | -0.18 | .002 |
| 2-4 times a month | 0.58 | .200 | 1.03 | .000 | -0.32 | .000 |
| 2-3 times a week | 0.98 | .042 | 1.59 | .000 | -0.32 | .000 |
| 4 or more times a week | 1.24 | .015 | 1.81 | .000 | -0.39 | .000 |
| Smoke (ref = never) |  |  |  |  |  |  |
| Current | -3.86 | .000 | -1.80 | .000 | 0.71 | .000 |
| Former | -0.94 | .002 | -0.45 | .006 | 0.10 | .021 |
| Comorbidity (ref =no) |  |  |  |  |  |  |
| COPD | -2.54 | .000 | -2.63 | .000 | 0.35 | .001 |
| Paralysis | 0.22 | .911 | -1.10 | .280 | 0.09 | .749 |
| Heart attack | -0.94 | .204 | -0.92 | .019 | 0.19 | .078 |
| Carotid artery disease | -0.93 | .524 | 0.21 | .791 | -0.17 | .427 |
| Stroke | -1.50 | .128 | -0.35 | .500 | 0.22 | .121 |
| Rheumatoid arthritis | -0.32 | .505 | -1.19 | .000 | 0.16 | .023 |
| Diabetes | 0.52 | .197 | -0.32 | .132 | 0.09 | .148 |
| Cancer | -0.61 | .246 | -0.02 | .944 | -0.09 | .251 |
| Liver disease | -1.07 | .302 | -0.49 | .373 | 0.05 | .747 |
| Peripheral vascular disease | 0.09 | .933 | -0.67 | .217 | -0.01 | .946 |
| Kidney disease | -1.83 | .030 | -1.46 | .001 | 0.14 | .269 |
| Ulcer disease | -1.72 | .093 | -1.02 | .057 | 0.34 | .023 |
| HIV or AIDS | 1.77 | .362 | 1.76 | .082 | 0.04 | .890 |
| Constant | 38.2 | .000 | 43.3 | .000 | 6.96 | .000 |
|  | $\beta$ | *SE* | $\beta$ | *SE* | $\beta$ | *SE* |
| Variance parameters |  |  |  |  |  |  |
| Center (se) | 1.13 | .515 | 0.44 | .190 | 0.06 | .024 |
| Participant (se) | 70.4 | 2.20 | 23.9 | .600 | 1.46 | .047 |
| Residual (se) | 121.8 | 1.56 | 23.4 | .290 | 3.02 | .037 |

Note: Estimated coefficients were from multivariable mixed effects regression. Standard errors were clustered for observations within individual patients and patients nested within hospitals.
